# Association of preoperative chronic opioid use with 1-year revision rate, mortality, and patient-reported outcomes after primary hip and knee arthroplasty: age, sex and BMI matter – a Dutch register-based study

**DOI:** 10.2340/17453674.2025.44597

**Published:** 2025-11-22

**Authors:** Heather E VAN BRUG, Rob G H H NELISSEN, Frits R ROSENDAAL, Liza N VAN STEENBERGEN, Marcel L BOUVY, Albert DAHAN, Maaike G J GADEMAN

**Affiliations:** 1 Department of Orthopaedics, Leiden University Medical Center, Leiden; 2 Department of Clinical Epidemiology, Leiden University Medical Center, Leiden; 3 Dutch Arthroplasty Register (LROI), ‘s Hertogenbosch; 4 Utrecht Institute for Pharmaceutical Sciences (UIPS), Division of Pharmacoepidemiology and Clinical Pharmacology, Utrecht University, Utrecht; 5 Department of Anaesthesiology, Leiden University Medical Center, Leiden, The Netherlands

## Abstract

**Background and purpose:**

Our aim was to study the association between chronic preoperative opioid use and 1-year revision rate, mortality, and patient-reported outcomes (PROs) after primary total knee and hip arthroplasty (TKA/THA). We also investigated whether age, sex, or BMI modified these associations.

**Methods:**

TKAs and THAs performed for osteoarthritis between 2013 and 2018, originating from the Dutch Arthroplasty Register, were linked to the Dutch Foundation for Pharmaceutical Statistics. Chronic preoperative opioid use was defined as > 1,800 morphine mg equivalent dispensed 1 year before surgery and ≥ 1 opioid prescribed 30 days before surgery. Outcomes were 1-year revision rate, mortality, self-reported physical functioning, pain, and quality of life (QoL). Incidence rates were calculated; Cox regression and linear mixed models were used. We assessed effect modification by assessment of supra-additive effects.

**Results:**

Preoperative chronic opioid use occurred in 4.5% of 29,739 THAs and 3.4% of 27,873 TKAs. Chronic opioid use doubled mortality and revision rates for both TKAs and THAs (range of hazard ratios 1.7–2.1). The association of preoperative opioid use with 1-year revision rate was larger in males, in patients with a BMI ≤ 30 (THA) and > 30 (TKA), and 66–75-year-olds. Younger patients exhibited a more pronounced association between opioid use and reduced physical functioning and QoL, and increased pain. Sex and BMI had no modifying effects on PROs.

**Conclusion:**

Preoperative chronic opioid use was associated with a higher likelihood of 1-year revision and mortality and worse PROs. The associations with revision risk were modified by age, sex, and BMI. Age also had a modifying effect on PROs.

Roughly 25% of the individuals with a total knee or hip arthroplasty (TKA/THA) receive opioids in the year before surgery [1], with increasing numbers in the Netherlands [2], although opioids seem to offer no advantage over placebo [3]. Consequently, international guidelines advise against their use [4] and opioid use is considered low-value care in osteoarthritis (OA) [5].

While establishing a causal relation is challenging, preoperative opioid use has been associated with poorer outcomes after TKA/THA. Studies suggest that opioid use before surgery results in a higher revision risk, and other outcomes such as pain and worse function [6-8]. These studies were all performed in the USA, where healthcare systems differ significantly from those in Europe, potentially limiting generalizability of their finding to the European population. Furthermore, variations in defining preoperative opioid use were observed across studies. Recently, a dose–response relationship was observed between preoperative opioid use and postoperative outcomes, underscoring the importance of distinguishing between chronic users and occasional or non-users [9]. Furthermore, studies investigating the effect of preoperative opioid use on mortality after arthroplasty are limited [10].

Chronic opioid use may affect postoperative outcomes differently across patient populations. Higher body mass index (BMI) and younger age have been associated with increased revision risks [11-13]. Also, postoperative patient-reported outcomes (PROs) like physical function and pain tend to be lower for women, elderly adults, and those with a high BMI [14,15], while opioid use is prevalent in these populations [16]. Hence, we aimed to examine the association between chronic preoperative opioid use and revision rate and mortality risk and its association with PROs. We also assessed whether sex, age, or BMI modify these potential associations.

## Methods

### Study design

We conducted a longitudinal cohort study in which we linked 2 national databases, the Dutch Arthroplasty Register (LROI) and the Dutch Foundation for Pharmaceutical Statistics (SFK). The study is reported according to STROBE guidelines.

### Data sources

The LROI covers all hospitals performing arthroplasties in the Netherlands. The data completeness of primary TKA and THA is > 98% [17]. The LROI provided information on individual arthroplasties, with patient and prosthesis characteristics. Pharmaceutical dispensing data was obtained from the SFK, containing data from > 95% of the community pharmacies and outpatient pharmacies within hospitals (these numbers are based on the SFK administration, the Royal Dutch Pharmacists Association [KNMP] administration and IGJ register of licensed pharmacists) [18]. Opioid dispensing data was derived 1 year before and 1 year after arthroplasty, including Anatomic-Therapeutic-Chemical (ATC) codes from the World Health Organization, the dose, the number dispensed, and information regarding the prescriber.

### Data linkage

Linkage and quality checks have previously been described in detail [19]. In short, the linkage between datasets was performed on a combination of year of birth, sex, 4-digit postcode, and surgery date together with the dispensing date of low-molecular-weight heparin prescribed around the surgery date (4 days before–10 days after) as a proxy for surgery date, which was unavailable in the SFK. Also, only arthroplasties that could be linked to dispensing data from a community pharmacy were included to be able to follow prescriptions over time.

### Study population

All linked first performed primary TKA and THA per patient (index arthroplasties) for OA between 2014 and 2018 were included. Exclusion criteria were patients younger than 18 years, arthroplasties with administrative errors (e.g., wrongly retrieved survival time), or > 4,000 defined daily dosages of opioids preoperatively.

### Preoperative chronic opioid use

Opioids were classified according to the ATC-5 classification. Opioid exposure was assessed as the total morphine milligram equivalent (MME), also “opioid dose” in the year before surgery. MMEs were calculated with the ATC classification by adding the dosages of the opioid prescription and multiplying this dose by an MME conversion factor based on the MME conversion table of the CDC (2014) [20]. For example, 1 mg oxycodone equals 1.5 MME, while 1 mg tramadol equals 0.1 MME. Preoperative chronic opioid use was defined as > 1,800 MMEs dispensed in the year before surgery and ≥ 1 opioid prescription in the month before surgery [21].

All patients not fulfilling the criteria for chronic opioid use were categorized in the group “others.”

### Outcomes after arthroplasty

Revision surgery was defined as any exchange (addition, replacement, or removal) of at least 1 component of the prosthesis within 1 year after surgery.

Mortality was defined as death within 1 year after arthroplasty.

Patient-reported outcomes (PROs) were the following: physical functioning, pain at rest and during activity, and quality of life (QoL). These PROs were collected using questionnaires before surgery and at 3 (TKA), 6 (THA), and 12 months postoperatively.

Physical functioning was assessed with the Hip disability or Knee Injury and Osteoarthritis Outcome Score – Physical Function Short form (HOOS-PS/KOOS-PS), consisting of, respectively, 5 and 7 items. The scores range from severe functional impairment (score 0) to no functional impairment (score 100). The Numeric Rating Scale (NRS) was used to assess pain during rest and activity in the last week, (0 = no pain and 10 = worst imaginable pain). The EuroQol 5-dimensions (EQ-5D-3L) questionnaire was used to measure health-related QoL, consisting of both the EQ-5D index score and the Visual Analogue Scale (VAS). The index score using the Dutch tariff ranges from –0.329 to 1 (worst imaginable health [worse than death]–best imaginable health) and the VAS from 0–100 [22].

### Effect modifiers: age, sex, and BMI

Age was assessed in age groups: ≤ 55, 56–65, 66–75 and, > 75 years old. Sex was categorized into males/females, BMI was classified as < 30 and ≥ 30.

### Patient characteristics

The following patient characteristics were available: current smoking status (yes/no), American Society of Anesthesiologists (ASA) classifications (ranging from I = healthy to IV = severe systemic disease, constant threat to life), and Charnley score. The Charnley score categorizes the degree of OA ranging from A = 1 joint affected to C = multiple joints affected or a chronic disease that limits QoL. Socioeconomic status (SES) was based on individual 4-digit postcodes and classified according to 2014–2016 measurements of the Netherlands Institute for Social Research, based on income, education, and occupation in quintile z-scores: very low (≤ –1.5), below average (–1.49 to –0.5), average (–0.49 to 0.49), above average (0.5–1.49), and very high (≥ 1.5).

### Statistics

Data cleaning was performed according to the guidelines of the LROI. Analyses were stratified for TKA and THA. Continuous outcomes were shown as mean with standard deviation (SD) and categorical outcomes as a proportion per category.

Rates of revision and mortality were calculated per 1,000 person-years (py) over 1-year follow-up separately for preoperative chronic opioid users and others (including non-users). Effect modification was assessed on the additive scale [23]. To assess effect modification, we calculated revision rates for different strata of the age, sex, and BMI groups and estimated the interaction contrasts based on the risk difference [24]. We constructed Cox proportional hazards models adjusted for sex, age, BMI, and ASA classification to determine whether preoperative chronic opioid use was associated with revision surgery and mortality.

To assess whether preoperative chronic opioid use is associated with physical functioning, pain, and QoL 1 year after arthroplasty, we conducted linear mixed-effect models (including subject-specific intercepts and slopes where model fit allowed). All models included an interaction term between time and opioid use. We conducted crude models as well as models adjusted for sex, age, BMI, and ASA classification. Model fit was evaluated using likelihood ratio tests (comparing random intercept vs random slope models) and visual inspection of residual plots (Q–Q plots). To assess effect modification, the linear mixed-effect models were separately fitted for the different prespecified strata of age, sex, and BMI. In addition, we added an interaction term to the models.

Missing data of the PROs were accounted for by the linear mixed models used. For all other variables, missing data was minimal and we conducted the analyses using available data without imputation

All statistical analyses were performed using the R software (version 4.2.1; R Foundation for Statistical Computing, Vienna, Austria).

### Ethics, registration, data sharing plan, funding, and disclosurest

Approval by a medical ethics committee was waived by the Medical Ethics Committee Leiden-Den Haag-Delft (G19.018). Data from both LROI and SFK was pseudonymized before they were received. Data cannot be shared publicly because of confidentiality. Data is available from the SFK and LROI (info@sfk.nl/ lroi@orthopeden.org) for researchers who meet the criteria for access to confidential data. This study was funded by the Van Rens Foundation (Project number: VRF2019-002). Grammar checking was performed using AI. There are no conflicts of interest for any of the authors. Complete disclosure of interest forms according to ICMJE are available on the article age, doi: 10.2340/17453674.2025.44597

## Results

### Population

**Figure 1:**
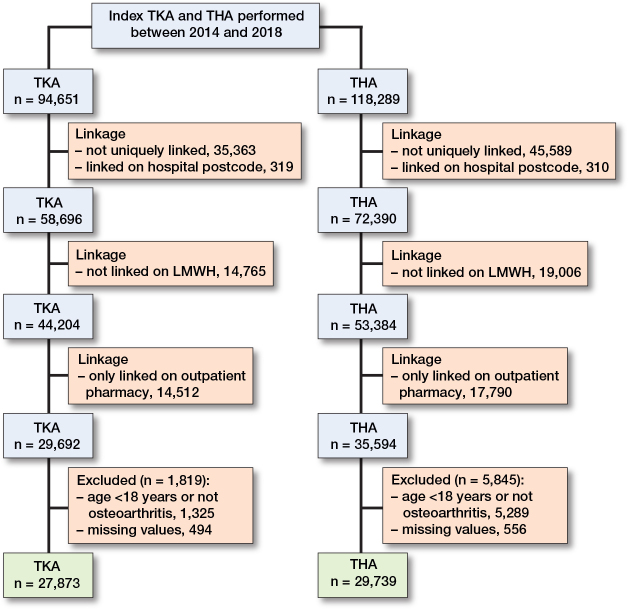
Patient flowchart. TKA: Total knee arthroplasty; THA: total hip arthroplasty; LMWH: low-molecular-weight heparin

Between 2014 and 2018, 94,651 index primary TKAs and 118,289 index primary THAs were performed. The study population consisted of 27,873 TKAs and 29,739 THAs, all performed for OA (Figure 1). Of these, 953 THAs (3%) and 1,339 TKAs (5%) were classified as preoperative chronic opioid users. At TKA, irrespective of preoperative opioid use, the mean age was around 67 years (Table 1). The proportion of females was higher among chronic preoperative users (72% vs 57%). Among the “others” group, 20% were prescribed at least 1 opioid in the year before surgery and 5% in the month before surgery. The median annual MME among preoperative chronic users was 5,145 MME (IQR 3,005–11,000) and for the opioid users in the “others” group, this was 300 MME (IQR 120–750).

**Table 1:** Characteristics of study population, preoperative chronic opioid users, and patients who did not chronically use opioids before surgery (including non-users). Values are count (%) unless otherwise specified

Factor	Knee arthroplasty		Hip arthroplasty	
	Chronic opioid users n = 953	Others n = 26,920	Chronic opioid users n = 1,339	Others n = 28,400
Demographics				
Age, mean (SD)	66.5 (10.1)	67.2 (9.0)	68 (11.1)	68.0 (9.9)
Age groups				
≤ 65	174 (18)	11,311 (42)	522 (39)	10,494 (37)
66–75	309 (32)	10,468 (39)	458 (34)	11,151 (39)
> 75	200 (21)	5,141 (19)	359 (27)	6,755 (24)
Female sex	687 (72)	15,461 (57)	886 (66)	16,902 (60)
BMI, mean (SD)	31.3 (5.7)	29.6 (4.9)	28.3 (5.2)	27.4 (4.4)
BMI groups				
< 30	419 (44)	15,949 (59)	891 (66)	21,720 (76)
≥ 30	534 (56)	10,971 (41)	448 (34)	6,680 (24)
ASA class				
I	49 (5.1)	4,075 (15)	87 (6.5)	5,719 (20)
II	580 (61)	18,475 (69)	833 (62)	18,606 (66)
III–IV	324 (34)	4,37 (16)	419 (31)	4,075 (14)
Charnley classification				
A	428 (45)	13,50 (50)	705 (53)	15,409 (54)
B1	413 (43)	10,86 (40)	449 (34)	9,710 (34)
B2	64 (6.7)	1,687 (6.0)	125 (9.3)	2,530 (8.8)
C	24 (2.5)	404 (1.5)	41 (3.1)	354 (1.2)
Not applicable/missing	24 (2.5)	467 (1.7)	19 (1.4)	397 (1.4)
SES				
Very low	153 (16)	3,675 (14)	212 (16)	3,131 (11)
Below average	195 (20)	5,113 (19)	270 (20)	5,126 (18)
Average/missing	370 (39)	10,284 (38)	493 (37)	10,829 (38)
Above average	196 (21)	6,585 (24)	302 (23)	7,690 (27)
Very high	39 (4.1)	1,263 (4.6)	62 (4.6)	1,624 (5.7)
Smoking	129 (14)	2,542 (9.4)	224 (17)	3,130 (12)
Missing	49 (5.1)	1,381 (5.1)	46 (3.4)	1,420 (5.0)
Preoperative opioid users				
1 year before surgery	953 (100)	5,458 (20)	1,339 (100)	6,240 (22)
MME, median	5,145	300	4,725	310
IQR lower	3,005	120	2,850	50
IQR upper	11,000	750	9,900	864
1 month before surgery	953 (100)	1,265 (4.6)	1,339 (100)	1,945 (6.8)
MME, median	864	150	900	210
IQR lower	450	113	450	113
IQR upper	1,800	300	1,800	360
Preoperative PROs, mean (SD)				
HOOS-PS/KOOS-PS	59 (16)	51 (15)	60 (18)	48 (18)
Missing, n (%)	640 (67)	18,350 (68)	820 (61)	16,692 (59)
Pain at rest NRS	68 (6)	5.1 (2.6)	6.4 (2.4)	5.0 (2.6)
Missing, n (%)	646 (68)	18,733 (70)	786 (59)	16,170 (57)
Pain while active NRS	8 (2)	7.2 (2.1)	8.1 (1.9)	7.0 (2.1)
Missing, n (%)	645 (68)	18,744 (70)	786 (59)	16,088 (57)
EQ-5D-3L index score	0.4 (0.2)	0.6 (0.2)	0.4 (0.2)	0.5 (0.2)
Missing, n (%)	633 (66)	18,326 (68)	786 (59)	16,111 (57)
EQ-5D VAS	58 (21)	68 (20)	56 (23)	66 (20)
Missing, n (%)	638 (67)	18,273 (68)	783 (58)	16,098 (57)

n: number of arthroplasties, SD: standard deviation, BMI: body mass index, ASA: American Society of Anesthesiologists Health Status Classification, SES: socioeconomic status, Charnley Classification A: 1 joint affected with osteoarthrosis; B1: 2 joints affected (both hips/both knees); B2: Contralateral joint with prosthesis; C: Multiple joints affected with osteoarthrosis or a chronic disease impairing quality of life (in walking). MME: total morphine milligram equivalent; HOOS-PS: Hip disability and Osteoarthritis Outcome Score – Physical Function Short Form; KOOS-PS: Knee injury and Osteoarthritis Outcome Score; NRS: Numeric Rating Scale score; EQ-5D: EuroQol 5 dimensions; EQ VAS: EuroQol Visual Analogue Scale.

In THA patients, the mean age in both groups was 68 years; chronic users were more often female than the “others” (66% vs 60%). Of patients in the “others” group 22% were prescribed at least 1 opioid in the year before surgery, and 7% in the month before surgery. The median annual MME among preoperative chronic users was 4,725 (IQR 2,850–9,900) and for the opioid users in the “others” group, this was 310 MME (IQR 150–864). Missingness for all variables is indicated in Table 1.

### Mortality

The 1-year mortality rate in TKA patients with chronic preoperative opioid use was 11.6/1,000 py, and 5.1/1,000 py for the “others” (Table 2). The adjusted hazard ratio (HR) for mortality in chronic opioid users was 2.1 (95% confidence interval [CI] 1.1–3.9). The 1-year mortality rate in THA patients with chronic preoperative use was 17.3/1,000 py and 7.5/1,000 py for the “others.” The adjusted HR for revision in chronic opioid users was 1.7 (CI 1.1–2.6).

**Table 2:** Preoperative chronic opioid use and revision and mortality 1 year after total knee arthroplasty and total hip arthroplasty

	n	Events n (%)	Rate / 1,000 py	Crude HR (CI)	Adjusted HR (CI) ^a^
Total knee arthroplasty					
Mortality					
Others	26,920	136 (0.5)	5.1	Ref.	Ref.
Preop. COU	953	11 (1.2)	11.6	2.3 (1.2–4.2)	2.1 (1.1–3.9)
Revision					
Others	26,920	303 (1.1)	11.4	Ref.	Ref.
Preop. COU	953	21 (2.2)	22.5	2.0 (1.2–3.1)	1.8 (1.2–2.9)
Total hip arthroplasty					
Mortality					
Others	28,400	212 (0.7)	7.5	Ref.	Ref.
Preop. COU	1,339	23 (1.7)	17.3	2.3 (1.5–3.6)	1.7 (1.1–2.6)
Revision					
Others	28,400	477 (1.7)	17.1	Ref.	Ref.
Preop. COU	1,399	48 (3.6)	37.3	2.2 (1.6–2.9)	2.0 (1.5–2.7)

CI: 95% confidence interval; COU: chronic opioid users; HR: hazard ratio; n: number of arthroplasties; py: person years. BMI: body mass index

a Adjusted for age, sex, BMI, and American Society for Anaesthesiologist Classification

### Revisions

The 1-year revision rate in TKA patients with preoperative chronic opioid use was 22.5/1,000 py and 11.4/1,000 py for “other’”TKA patients. The adjusted HR for revision was 1.8 (CI 1.2–2.9). The 1-year revision rate in THA patients with preoperative chronic use was 37.3/1,000 py and 17.1/1,000 py in the “others.” The adjusted HR was 2.0 (CI 1.5–2.7) (see Table 2).

#### Effect modification by sex, age, and BMI

**Figure 2:**
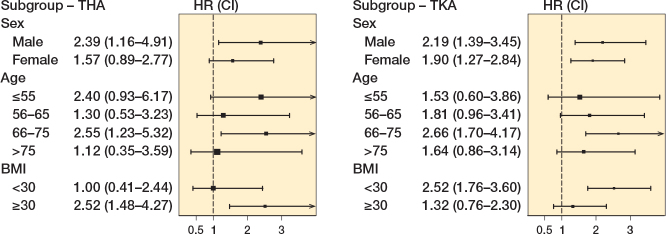
Association between chronic opioid usage and 1-year revision rate in different strata based on sex, age, and body mass index for (left panel) total hip arthroplasty (THA) and (right panel) total knee arthroplasty (TKA). CI: 95% confidence interval; HR: hazard ratio.

We observed effect modification on the incidence rate for revision by age, sex, and BMI (Figure 2 and Table 3). Superadditive effects were observed between male sex and chronic preoperative opioid use for both TKA (excess risk = 11.5/1,000py) and TKA (excess risk = 17.1/1,000py), indicating that male chronic opioid users had a higher risk of revision compared with the sum of individual effects. The association of chronic opioid use was also higher in TKA patients with a BMI > 30 than in patients with a BMI ≤ 30 (mean difference: 19.2/1,000 py) and in THA patients with a BMI ≤ 30 than in patients with BMI > 30 (mean difference: 14.4/1,000 py). The association of chronic opioid use with revision risk was higher in THA patients aged 56–65 and 66–75 years than in patients in other age groups (Table 3). Among TKA patients the association of chronic opioid use was higher in the age groups < 55 and 66–75 years old than in patients in other age groups.  

**Table 3:** Incidence rates of revision arthroplasty by preoperative chronic use status in different patient groups

Chronic opioid use	Patient group	Revision IR (/1,000 py)
Total knee arthroplasty		
No	Male sex	11.5 (11.5–11.5)
No	Female sex	11.3 (11.3–11.3)
Yes	Male sex	30.9 (30.9–31.0)
Yes	Female sex	19.3 (19.3–19.3)
No	BMI ≤ 30	11.6 (11.6–11.6)
No	BMI > 30	11.0 (11.0–11.0)
Yes	BMI ≤ 30	12.1 (12.1–12.2)
Yes	BMI > 30	30.7 (30.7–30.8)
No	Age < 55	14.7 (14.7–14.8)
No	Age 56–65	11.9 (11.9–11.9)
No	Age 66–75	9.4 (9.4–9.4)
No	Age > 75	12.6 (12.6–12.7)
Yes	Age < 55	35.6 (35.6–35.7)
Yes	Age 56–65	16.9 (16.9–17.0)
Yes	Age 66–75	26.5 (26.4–26.6)
Yes	Age > 75	15.4 (15.3–15.4)
Total hip arthroplasty		
No	Male	20.4 (20.4–20.4)
No	Female	14.8 (14.8–14.8)
Yes	Male	48.7 (48.6–48.7)
Yes	Female	31.5 (31.5–31.6)
No	BMI ≤ 30	15.4 (15.4–15.4)
No	BMI > 30	22.5 (22.5–22.5)
Yes	BMI ≤ 30	39.7 (39.7–39.8)
Yes	BMI > 30	32.4 (32.4–32.4)
No	Age < 55	16.9 (16.9–16.9)
No	Age 56–65	16.1 (16.1–16.1)
No	Age 66–75	17.1 (17.0–17.1)
No	Age > 75	18.4 (18.3–18.4)
Yes	Age < 55	27.3 (27.2–27.4)
Yes	Age 56–65	33.9 (33.8–33.9)
Yes	Age 66–75	50.3 (50.3–50.4)
Yes	Age > 75	29.2 (29.1–29.2)

IR: incidence rate; BMI: body mass index.

### Physical functioning, pain, and QoL

Only the adjusted models are shown; no large differences between crude and adjusted models were found. Overall, for both TKA and THA patients, the group with chronic opioid use reported worse physical functioning, pain and QoL before surgery compared with the “other” patients.

**Figure 3:**
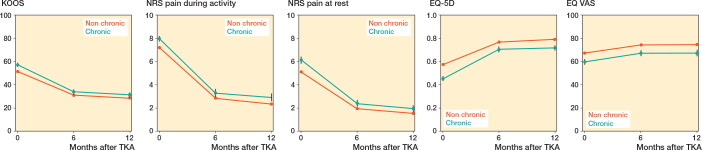
Patient-reported outcome measures for total knee arthroplasties over time based on mixed-effect model analyses. 0: indicating preoperative measurement; 6, 12: measurement months postoperatively. KOOS-PS: Knee injury and Osteoarthritis Outcome Score; NRS: Numeric Rating Score; EQ-5D: EuroQol 5 dimensions; EQ VAS: EuroQol Visual Analogue Scale.

**Figure 4:**
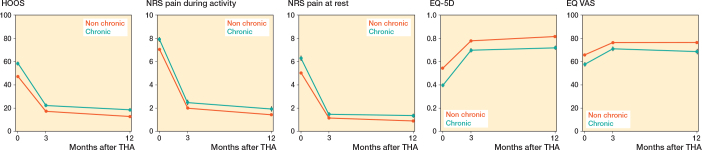
Patient-reported outcome measures for total hip arthroplasties over time based on mixed-effect model analyses. 0: indicating preoperative measurement; 3, 12: measurement months postoperatively. HOOS-PS: Hip disability and Osteoarthritis Outcome Score – Physical Function Short Form; NRS: Numeric Rating Scale score; EQ-5D: EuroQol 5 dimensions; EQ VAS: EuroQol Visual Analogue Scale.

Although these outcomes improved over time for both groups after arthroplasty, the chronic opioid group showed greater improvement at 6 months and 1 year postoperatively; however, mean scores remained lower than those of the “others” group (Figures 3–4, Tables 4–5).

**Table 4:** Models showing the associations between preoperative chronic opioid use and several patient-reported outcomes for total knee arthroplasties

TKA	KOOS-PS n = 10,968	NRS Pain while active n = 10,549	NRS Pain at rest n = 10,549	EQ-5D-3L n = 11,004	EQ VAS n = 11,029
Model 1: Overall					
Chronic opioid use	5.87 (4.23 to 7.52)	0.77 (0.54 to 1.00)	1.04 (0.74 to 1.33)	–0.12 (–0.14 to –0.10)	–7.73 (–9.97 to –5.48)
Chronic opioid use*FU6	–3.02 (–5.30 to –0.73)	–0.33 (–0.72 to 0.06)	–0.60 (–0.98 to –0.21)	0.06 (0.03 to 0.08)	0.64 (–2.43 to 3.72)
Chronic opioid use*FU12	–2.98 (–5.34 to –0.62)	–0.19 (–0.58 to 0.20)	–0.61 (–0.99 to –0.23)	0.05 (0.02 to 0.07)	0.40 (–2.79 to 3.59)
Model 2: Age					
Chronic opioid use	4.93 (0.50 to 9.37)	0.62 (–0.03 to 1.27)	0.30 (–0.53 to 1.12)	–0.11 (–0.17 to –0.06)	–6.90 (–13.1 to –0.76)
Chronic opioid use*FU6	–1.94 (–3.06 to –0.81)	–0.03 (–1.14 to 1.07)	0.45 (–0.67 to 1.56)	0.04 (–0.04 to 0.12)	1.42 (–7.36 to 10.2)
Chronic opioid use*FU12	–4.67 (–5.78 to –3.56)	0.40 (–0.77 to 1.56)	0.67 (–0.48 to 1.81)	–0.02 (–0.10 to 0.05)	–3.96 (–13.6 to 5.72)
Age					
< 55	Ref	Ref	Ref	Ref	Ref
56–65	–1.94 (–3.06 to –0.81)	–0.27 (–0.43 to –0.11)	–0.54 (–0.75 to –0.34)	0.04 (0.03 to 0.06)	1.86 (0.33 to 3.39)
66–75	–4.67 (–5.78 to –3.56)	–0.62 (–0.78 to –0.47)	–1.04 (–1.24 to –0.84)	0.07 (0.06 to 0.09)	5.34 (3.83 to 6.85)
> 75	–2.97 (–4.21 to –1.72)	–0.65 (–0.83 to –0.47)	–1.36 (–1.58 to –1.13)	0.05 (0.04 to 0.07)	4.74 (3.04 to 6.43)
Age*chronic opioid use					
< 55*CPO	Ref	Ref	Ref	Ref	Ref
56–65*CPO	0.20 (–5.05 to 5.46)	0.17 (–0.59 to 0.93)	0.81 (–0.16 to 1.77)	–0.01 (–0.07 to 0.05)	0.33 (–6.92 to 7.58)
66–75*CPO	3.11 (–2.11 to 8.33)	0.06 (–0.69 to 0.82)	0.83 (–0.12 to 1.79)	–0.01 (–0.07 to 0.05)	–0.88 (–8.07 to 6.31)
> 75*CPO	–1.80 (–7.70 to 4.10)	0.35 (–0.49 to 1.19)	0.81 (–0.26 to 1.87)	0.01 (–0.06 to 0.08)	–2.48 (–10.6 to 5.65)
Age*chronic opioid use*time					
56–65*CPO*FU6	0.48 (–7.08 to 8.05)	–0.57 (–1.85 to 0.72)	–1.20 (–2.48 to 0.08)	0.04 (–0.06 to 0.13)	–0.37 (–10.6 to 9.8)
56–65*CPO*FU12	–3.98 (–12.10 to 4.14)	–1.00 (–2.34 to 0.33)	–1.76 (–3.07 to –0.45)	0.12 (0.03 to 0.21)	4.97 (–6.09 to 16.0)
66–75*CPO*FU6	–1.33 (–8.93 to 6.27)	–0.12 (–1.40 to 1.17)	–1.13 (–2.41 to 0.15)	0.02 (–0.07 to 0.11)	–0.54 (–10.7 to 9.63)
66–75*CPO*FU12	–4.22 (–12.31 to 3.87)	–0.25 (–1.57 to 1.07)	–1.18 (–2.48 to 0.13)	0.04 (–0.05 to 0.13)	3.43 (–7.55 to 14.4)
> 75*CPO*FU6	0.99 (–7.68 to 9.67)	–0.36 (–1.84 to 1.11)	–1.19 (–2.65 to 0.27)	–0.02 (–0.12 to 0.09)	–3.47 (–15.1 to 8.14)
> 75*CPO*FU12	–3.90 (–13.3 to 5.53)	–0.88 (–2.40 to 0.65)	–1.18 (–2.67 to 0.31)	0.08 (–0.02 to 0.18)	6.59 (–6.02 to 19.21)
Model 3: Sex					
Chronic opioid use	8.07 (5.17 to 10.97)	1.16 (0.75 to 1.57)	1.37 (0.85 to 1.89)	–0.12 (–0.16 to –0.09)	–4.73 (–8.86 to –0.78)
Chronic opioid use*FU6	–3.85 (–7.86 to 0.17)	–0.46 (–1.15 to 0.23)	–0.93 (–1.61 to –0.25)	0.06 (0.01 to 0.11)	–3.35 (–8.80 to 2.10)
Chronic opioid use*FU12	–2.18 (–6.32 to 1.95)	–0.43 (–1.12 to 0.25)	–0.84 (–1.51 to –0.16)	0.05 (0.00 to 0.09)	–3.51 (–9.16 to 2.13)
Female	4.57 (3.95 to 5.19)	0.38 (0.29 to 0.47)	0.59 (0.48 to 0.70)	–0.00 (–0.00 to –0.00)	–3.51 (–4.53 to –2.66)
Female*CPO	–3.35 (–6.86 to 0.16)	–0.57 (–1.06 to –0.08)	–0.53 (–1.15 to 0.10)	0.01 (–0.04 to 0.05)	–4.36 (–9.15 to 0.43)
Female*CPO*FU6	1.61 (–3.27 to 6.49)	0.18 (–0.65 to 1.02)	0.55 (–0.27 to 1.37)	–0.01 (–0.07 to 0.05)	5.79 (–0.82 to 12.4)
Female*CPO*FU12	–0.90 (–5.93 to 4.14)	0.37 (–0.47 to 1.20)	0.40 (–0.41 to 1.22)	–0.00 (–0.06 to 0.05)	5.62 (–1.23 to 12.5)
Model 4: BMI					
Chronic opioid use	5.10 (2.60 to 7.60)	0.93 (0.58 to 1.28)	1.11 (0.67 to 1.54)	–0.10 (–0.13 to –0.07)	–10.4 (–13.8 to –7.02)
Chronic opioid use*FU6	–1.18 (–4.57 to 2.21)	–0.21 (–0.78 to 0.37)	–0.52 (–1.09 to 0.05)	0.02 (–0.01 to 0.06)	3.68 (–0.85 to 8.22)
Chronic opioid use*FU12	–1.64 (–5.28 to 2.01)	–0.25 (–0.85 to 0.34)	–0.36 (–0.94 to 0.23)	0.02 (–0.02 to 0.06)	1.19 (–3.69 to 6.07)
BMI > 30	3.52 (2.87 to 4.16)	0.26 (0.17 to 0.35)	0.42 (0.31 to 0.54)	–0.03 (–0.04 to –0.02)	–2.01 (–2.89 to –1.14)
BMI > 30*CPO	1.30 (–2.02 to 4.61)	–0.29 (–0.75 to 0.18)	–0.16 (–0.75 to 0.42)	–0.03 (–0.07 to 0.01)	4.72 (0.22 to 9.23)
BMI > 30*CPO*FU6	–3.00 (–7.59 to 1.59)	–0.24 (–1.02 to 0.54)	–0.09 (–0.86 to 0.68)	0.06 (0.00 to 0.11)	–5.55 (–11.7 to 0.63)
BMI > 30*CPO*FU12	–2.22 (–7.01 to 2.57)	0.08 (–0.71 to 0.86)	–0.36 (–1.13 to 0.41)	0.04 (–0.01 to 0.10)	–1.54 (–8.00 to 4.92)

Adjusted for age, sex, BMI, and ASA classification.

TKA: total knee arthroplasty; KOOS-PS: Knee injury and Osteoarthritis Outcome Score – Physical Function Shortform; FU: follow-up in months; NRS: Numeric Rating Scale score; EQ: EuroQol; VAS: Visual Analogue Scale; ASA: American Society of Anesthesiologists; BMI: body mass index; CPO: Chronic preoperative opioid use.

**Table 5:** Models showing the associations between preoperative chronic opioid use and several patient-reported outcomes for total hip arthroplasties

THA	HOOS-PS n = 14,438	NRS Pain while active n = 14,845	NRS Pain at rest n = 14,761	EQ-5D-3L n = 14,905	EQ VAS n = 14,927
Model 1: Overall					
Chronic opioid use	11.0 (9.49 to 12.52)	0.88 (0.70 to 1.06)	1.25 (1.03 to 1.46)	–0.15 (–0.16 to –0.13)	–8.03 (–9.73 to –6.32)
Chronic opioid use*FU6	–5.92 (–7.86 to –3.98)	–0.39 (–0.66 to –0.12)	–0.93 (–1.19 to –0.67)	0.07 (0.05 to 0.09)	2.63 (0.37 to 4.88)
Chronic opioid use*FU12	–5.25 (–7.24 to –3.26)	–0.39 (–0.66 to –0.12)	–0.78 (–1.05 to –0.51)	0.05 (0.03 to 0.07)	0.17 (–2.31 to 2.65)
Model 2: Age					
Chronic opioid use	6.84 (3.18 to 10.50)	0.75 (0.31 to 1.18)	1.11 (0.58 to 1.64)	–0.15 (–0.19 to 0.12)	–9.44 (–13.6 to –5.29)
Chronic opioid use*FU6	–2.47 (–7.09 to 2.14)	–0.26 (–0.92 to 0.40)	–4.33 (–4.50 to –4.17)	0.05 (0.00 to 0.10)	2.30 (–3.15 to 7.75)
Chronic opioid use*FU12	3.88 (–1.06 to 8.82)	0.39 (–0.31 to 1.09)	–4.61 (–4.77 to –4.45)	–0.01 (–0.07 to 0.04)	–3.27 (–9.53 to 3.00)
Age					
< 55	Ref	Ref	Ref	Ref	Ref
56–65	–2.25 (–3.34 to –1.16)	–0.25 (–0.38 to –0.12)	–0.45 (–0.61 to –0.29)	0.05 (0.03 to 0.06)	3.44 (2.18 to 4.69)
66–75	–4.23 (–5.28 to –3.18)	–0.56 (–0.69 to –0.44)	–0.84 (–0.99 to –0.68)	0.07 (0.06 to 0.08)	6.50 (5.29 to 7.70)
> 75	–2.78 (–3.93 to –1.62)	–0.51 (–0.65 to –0.38)	–1.06 (–1.23 to –0.90)	0.05 (0.04 to 0.06)	6.30 (4.98 to 7.62)
Age*chronic opioid use					
< 55*CPO	Ref	Ref	Ref	Ref	Ref
56–65*CPO	4.03 (–0.75 to 8.81)	–0.02 (–0.58 to 0.55)	–0.01 (–0.70 to 0.68)	0.04 (–0.01 to 0.09)	3.33 (–2.07 to 8.74)
66–75*CPO	3.77 (–0.64 to 8.19)	0.11 (–0.41 to 0.64)	0.17 (–0.46 to 0.81)	0.01 (–0.04 to 0.05)	0.07 (–4.92 to 5.06)
> 75*CPO	7.65 (2.77 to 12.53)	0.38 (–0.19 to 0.96)	0.22 (–0.48 to 0.92)	–0.01 (–0.06 to 0.04)	3.66 (–1.83 to 9.15)
Age*chronic opioid use*time					
56–65*CPO*FU6	–5.88 (–11.9 to 0.12)	–0.07 (–0.92 to 0.79)	–0.21 (–1.04 to 0.61)	–0.00 (–0.07 to 0.06)	–0.39 (–7.45 to 6.66)
56–65*CPO*FU12	–11.6 (–18.0 to –5.29)	–0.42 (–1.31 to 0.47)	–0.25 (–1.11 to 0.61)	0.05 (–0.02 to 0.12)	–0.14 (–8.12 to 7.85)
66–75*CPO*FU6	–1.73 (–7.35 to 3.88)	0.10 (–0.70 to 0.90)	–0.61 (–1.41 to 0.19)	0.01 (–0.05 to 0.07)	–0.50 (–7.11 to 6.11)
66–75*CPO*FU12	–10.7 (–16.6 to –4.80)	–0.98 (–1.81 to –0.15)	0.07 (–0.70 to 0.84)	0.08 (0.02 to 0.15)	7.24 (–0.18 to 14.7)
> 75*CPO*FU6	–6.38 (–12.7 to –0.05)	–0.69 (–1.57 to 0.19)	–0.29 (–1.14 to 0.56)	0.05 (–0.02 to 0.11)	2.08 (–5.24 to 9.41)
> 75*CPO*FU12	–9.42 (–16.0 to –2.81)	–1.36 (–2.27 to –0.45)	–0.57 (–1.45 to 0.31)	0.08 (0.01 to 0.15)	2.37 (–5.88 to 10.6)
Model 3: Sex					
Chronic opioid use	11.6 (9.10 to 14.06)	0.93 (0.64 to 1.23)	1.40 (1.04 to 1.76)	–0.17 (–0.19 to –0.14)	–11.0 (–13.8 to –8.15)
Chronic opioid use*FU6	–5.29 (–8.45 to –2.14)	–0.16 (–0.60 to 0.28)	–0.98 (–1.41 to –0.56)	0.06 (0.03 to 0.09)	4.31 (0.63 to 8.00)
Chronic opioid use*FU12	–5.87 (–9.10 to –2.64)	–0.23 (–0.68 to 0.22)	–0.88 (–1.32 to –0.44)	0.05 (0.01 to 0.08)	3.82 (–0.28 to 7.92)
Female	4.48 (3.85 to 5.11)	0.46 (0.38 to 0.53)	0.62 (0.53 to 0.72)	–0.05 (–0.05 to –0.04)	–3.98 (–4.70 to –3.26)
Female*CPO	–0.99 (–4.12 to 2.14)	–0.09 (–0.46 to 0.28)	–0.26 (–0.71 to 0.19)	0.03 (0.00 to 0.06)	4.65 (1.12 to 8.19)
Female*CPO*FU6	–0.93 (–4.93 to 3.06)	–0.37 (–0.92 to 0.19)	0.10 (–0.44 to 0.65)	0.01 (–0.03 to 0.05)	–2.61 (–7.27 to 2.05)
Female*CPO*FU12	1.17 (–2.93 to 5.28)	–0.24 (–0.80 to 0.33)	0.19 (–0.37 to 0.74)	0.00 (–0.04 to 0.05)	–5.77 (–10.9 to –0.62)
Model 4: BMI					
Chronic opioid use	11.7 (9.87 to 13.60)	0.94 (0.72 to 1.16)	1.31 (1.05 to 1.58)	–0.15 (–0.17 to –0.13)	–8.36 (–10.5 to –6.26)
Chronic opioid use*FU6	–29.9 (–30.4 to –29.4)	–0.32 (–0.65 to 0.01)	–0.84 (–1.16 to –0.52)	0.07 (0.05 to 0.10)	1.49 (–1.27 to 4.25)
Chronic opioid use*FU12	–34.4 (–34.8 to –33.9)	–0.43 (–0.76 to –0.09)	–0.93 (–1.26 to –0.61)	0.05 (0.03 to 0.08)	1.13 (–1.91 to 4.16)
BMI > 30	3.27 (2.52 to 4.01)	0.28 (0.19 to 0.37)	0.25 (0.15 to 0.36)	–0.03 (–0.04 to –0.03)	–2.66 (–3.50 to –1.82)
BMI > 30*CPO	–2.47 (–5.67 to 0.74)	–0.22 (–0.60 to 0.16)	–0.25 (–0.71 to 0.21)	0.01 (–0.03 to 0.04)	1.22 (–2.38 to 4.82)
BMI > 30*CPO*FU6	–0.89 (–5.00 to 3.22)	–0.15 (–0.72 to 0.43)	–0.18 (–0.74 to 0.38)	–0.02 (–0.07 to 0.02)	2.92 (–1.88 to 7.71)
BMI > 30*CPO*FU12	3.58 (–0.65 to 7.80)	0.17 (–0.41 to 0.76)	0.54 (–0.03 to 1.12)	–0.01 (–0.05 to 0.04)	–3.12 (–8.39 to 2.14)

Adjusted for age, sex, BMI, and ASA classification.

THA: total hip arthroplasty; HOOS-PS: Hip disability and Osteoarthritis Outcome Score – Physical Function Shortform.

For abbreviations, also see Table 4.

#### Effect modification by sex, age, and BMI

After stratification, we again observed that all patients with preoperative chronic opioid use, irrespective of stratum, performed worse on physical functioning, pain, and QoL than the “others.” For TKA patients we observed effect modification by age and little effect modification by sex and BMI (see Tables 4–5). At 12 months, the association of chronic opioid use was larger in patients aged between 56 and 65 years, who improved more than the patients ≤ 55 years old on pain at rest and QoL (EQ-5D-3L).

For THA patients we observed effect modification by sex and age but not by BMI. Females with chronic opioid use improved less than males with chronic opioid use on QoL at 12 months (EQ VAS).

The group > 75 years old exhibited fewer negative effects of chronic opioid use on physical functioning than the ≤ 55-year-olds at 6 months.

At 12 months, compared with the group aged ≤ 55, all older age groups experienced smaller negative effects of chronic opioid use; they improved more on physical function. Also, the age groups > 66 years old experienced a smaller negative effect of chronic opioid use; they improved more on pain score during activity and QoL than patients ≤ 55 years old.

## Discussion

We aimed to examine the association between chronic preoperative opioid use in TKA and THA and (i) revision and mortality risk and (ii) its association with PROs and also assessed whether sex, age, or BMI modifies these potential associations. We found that preoperative chronic opioid use was associated with an increased risk of 1-year revision and mortality after TKA and THA. Patients’ age, being male, and BMI modified the association between preoperative chronic opioid use and revision risk after TKA and THA on the additive scale. In addition, preoperative chronic opioid use was also associated with worse physical functioning, more pain, and a lower QoL after both TKA and THA. Age modified the effect of preoperative use on physical functioning, pain, and QoL while sex and BMI had less pronounced effects on the PROs.

In contrast to our findings on mortality and preoperative chronic opioid use, others found no difference between TKA patients with chronic opioid use and opioid-naive patients in in-hospital mortality, and mortality at 30, 60, and 90 days postoperatively [10]. This may be explained by the longer follow-up in our study as we investigated 1-year mortality. Our finding, that preoperative chronic opioid use is associated with an increased revision risk, seems consistent with meta-analyses [6,7,25], although studies included in these meta-analyses did not consider the time to revision surgery. The hazard ratio, as the outcome of a time-to-event analysis, provides information on the instantaneous risk of revision at any given time, here in the first postoperative year. This makes it a more informative measure from a clinical perspective. Also, instead of comparing opioid users with opioid-naive patients, we compared preoperative chronic opioid users with the rest of the patients. We found that 5–7% of the TKA and THA patients in the latter group were prescribed an opioid 1 month before surgery. The similar associations found in our study underscore the importance of identifying chronic opioid use as a risk factor for worse outcomes. Furthermore, this distinction also better reflects clinical practice and enables the identification of possible targets for preventive strategies.

With regard to preoperative opioid use and postoperative PROs, data from a meta-analysis focusing on PROs showed that preoperative opioid users had worse postoperative PROs [8], which was in line with our findings. In our study, the association was present across all measured PROs, namely pain, physical functioning, and overall QoL.

Effect modification was observed in both the associations with revision surgery and the PROs. For revision surgery, the negative association of chronic opioid use and revision risk was stronger in males than in females. This aligns with previous registry-based findings demonstrating sex-based differences in revision risk after arthroplasty, with men generally having a higher risk of revision for infection compared with women [26]. The modifying effect of BMI was less clear. In TKA patients, those with a higher BMI and chronic opioid use had the highest revision risk whereas in THA patients the associated effect of chronic opioid use was largest in patients with a BMI ≤ 30. Age had a more diffuse association, as the effect modification differed over age groups. In our analysis of the PROs, we observed effect modification, primarily by age; specifically, older age groups showed a smaller adverse impact of preoperative opioid use compared with the cohort under the age of 55. These findings align with previous studies indicating that younger age is associated with both increased opioid use and less favorable PROs following arthroplasty [27, 28]. Moreover, effect modification is less frequently observed in younger patients [29]. Sex had only a modifying effect on QoL; here women who were preoperatively chronically exposed to opioids experienced a lower QoL. This differed from the effect modification by sex on revision surgery, where the association of chronic opioid use was largest in males. To our knowledge, no other studies have investigated age, sex, and BMI as effect modifiers in the association between preoperative opioid use and several postoperative outcomes. Our results indicate that chronic preoperative opioid use has an impact on worse outcomes after surgery. It remains uncertain whether this is a causal effect or simply a reflection of disparities in baseline risk, as stated by other studies [10,30]. Nevertheless, preoperative opioid prescriptions are deemed as low-value care and should therefore be limited [4]. Even more, Bloom et al. showed that the risk of worse outcomes in TKA/THA can be reduced without affecting patient satisfaction when prescribers adhere more strictly to guidelines on opioid use [31].

### Strength

A strength of our study is that we calculated MMEs allowing for a precise definition of chronic opioid use. Furthermore, we used 2 national datasets, which ensures national coverage.

### Limitations

First, dispensing data were used as a measure of opioid consumption, while the dispensed drug may not have been used. This could have introduced information bias by misclassifying exposure status. Second, concerning the statistical power, is that the number of revision surgeries may have been too low to analyze effect modification or explore associations with specific revision types. The calculated incidence rates suggest effect modification, which should be further investigated. Third, in relation to the PRO scores, much data was missing. To counter this, we used linear mixed models, allowing us to assess the PRO scores in a larger population. Furthermore, in cohort studies, residual confounding could always remain an issue. Fourth, concerning our data linkage, the datasets were not linked on a unique identifier, but on a combination of identifiers, resulting in some remaining uncertainty. In addition, approximately 30% of all index surgeries could be linked and therefore included in the analyses. Differences between groups seemed small but our linked population had a higher number of knee replacements, was a bit younger, had fewer females, more frequently had OA as the reason for surgery, and had slightly lower Charnley Scores and ASA classifications compared with the unlinked group [19]. However, this could possibly limit the generalizability of our findings. Further data linkage limitations have been published elsewhere [19]. Finally, we did not have information on the indication for opioid prescriptions, hence, some patients may have received opioids for reasons unrelated to the surgery.

### Conclusion

We observed that preoperative chronic opioid use was associated with higher 1-year revision rate and mortality risk and worse PROs in THA and TKA patients. Age, sex, and BMI all modified the effects of preoperative chronic opioid use on revision rate. Age also seemed to modify the effect of chronic preoperative opioid use on several PRO scores.

*In perspective,* preoperative chronic opioid use is a risk factor for worse outcomes and should therefore be limited, which necessitates assessment of opioid use before surgery. Even without causality, chronic preoperative opioids can still be used as a marker for poor postoperative outcomes and, if used, should warrant closer postoperative monitoring of these patients. Ultimately, prioritizing patient safety and outcomes requires assessing opioid use preoperatively, possibly weaning patients off opioids, and using chronic use as a marker for further close postoperative monitoring of these patients.

## Notes

Writing of the manuscript: HvB, MG. Conceptualizations and funding acquisition: RN, AD, MG. Data curation for the LROI data: LvS. Formal analyses and visualization: HvB. Methodology: HvB, RN, FR, MB, MG. Supervision: RN, MG. All authors interpreted the data and contributed to the final manuscript.

Handling co-editor: Per Henrik Randsborg

*Acta* thanks Thomas Jakobsen and Kevin Heebøll Nygaard for help with peer review of this manuscript.
